# Preparation of NiO NWs by Thermal Oxidation for Highly Selective Gas-Sensing Applications

**DOI:** 10.3390/s25072075

**Published:** 2025-03-26

**Authors:** Marwa Ben Arbia, Sung-Ho Kim, Jun-Bo Yoon, Elisabetta Comini

**Affiliations:** 1 Sensor Laboratory, Dipartimento di Ingegneria dell’Informazione, Università degli Studi di Brescia, Via Valotti 9, 25123 Brescia, Italy; m.benarbia@studenti.unibs.it; 2 School of Electrical Engineering, Korea Advanced Institute of Science and Technology (KAIST), 291 Daehak-ro, Yuseong-gu, Daejeon 34141, Republic of Korea; sungho05@kaist.ac.kr (S.-H.K.); jbyoon@kaist.ac.kr (J.-B.Y.)

**Keywords:** NiO NWs, thermal oxidation, gas sensing, NO_2_ selectivity

## Abstract

This paper presents a novel approach for fabricating porous NiO films decorated with nanowires, achieved through sputtering followed by thermal oxidation of a metallic layer. Notably, we successfully fabricate NiO nanowires using this simple and cost-effective method, demonstrating its potential applicability in the gas-sensing field. Furthermore, by using the film of our nanowires, we are able to easily prepare NiO sensors and deposit the required Pt electrodes directly on the film. This is a key advantage, as it simplifies the fabrication process and makes it easier to integrate the sensors into practical gas-sensing devices without the need for nanostructure transfer or intricate setups. Scanning electron microscopy (SEM) reveals the porous structure and nanowire formation, while X-ray diffraction (XRD) confirms the presence of the NiO phase. As a preliminary investigation, the gas-sensing properties of NiO films with varying thicknesses were evaluated at different operating temperatures. The results indicate that thinner layers exhibit superior performances. Gas measurements confirm the p-type nature of the NiO samples, with sensors showing high responsiveness and selectivity toward NO_2_ at an optimal temperature of 200 °C. However, incomplete recovery is observed due to the high binding energy of NO_2_ molecules. At higher temperatures, sufficient activation energy enables a full sensor recovery but with reduced response. The paper discusses the adsorption–desorption reaction mechanisms on the NiO surface, examines how moisture impacts the enhanced responsiveness of Pt-NiO (2700%) and Au-NiO (400%) sensors, and highlights the successful fabrication of NiO nanowires through a simple and cost-effective method, presenting a promising alternative to more complex approaches.

## 1. Introduction

High-performance gas sensors are attracting significant interest due to their wide-ranging applications in areas like chemical detection, medical and healthcare industries, and food processing and packaging. They allow for the controlling of pollution, the monitoring of gas concentrations and the ensuring of safety. Daily, the emission of air pollutants from sources like combustion engines and automobiles continues to rise. Among these, NO_2_ is one of the prevalent pollutants in the atmosphere, contributing to environmental issues such as acid rain, depletion of the ozone layer, and various respiratory diseases. Long-term exposure can contribute to the development of asthma and can make individuals more susceptible to respiratory infections. NO_2_, along with other nitrogen oxides (NO_x_), also contributes to the formation of harmful particulate matter and ozone, as well as acid rain, which damages ecosystems. Therefore, developing an efficient NO_2_ sensor is crucial for environmental monitoring and taking care of human health against excessive exposure. Traditional gas sensors for NO_2_ detection have utilized materials such as metal oxides (SnO_2_, ZnO, WO_3_, TiO_2_, Fe_2_O_3_, V_2_O_5_, CuO, NiO, etc.). Usually, metal oxides must be nanostructured to enhance the transducer function where controlling the morphology, the size, and the crystallinity can directly influence the carrier transport and, thus, the sensing response. Nanowires (NWs) are well recognized for this purpose as they limit conduction pathways along their axes and reduce the scattering phenomenon on the grain boundaries [1]. The design of single NWs provides a well-defined and reproducible path for charge carriers. This results in higher sensitivity in detecting gas molecules. A single tin oxide nanowire (60 nm in diameter, 3.5 μm long) is reported in reference [2] for a gas-sensing application, showing a good recognition of gases with a precision of 94.3% and a very low limit of detection (LOD < 1 ppm). The effect of a SnO_2_ nanowire’s size is studied in reference [3], which proves the depletion layer model. Compared to vertically aligned nanowires that have issues with uniformity and alignment precision over large areas, single nanowires can be individually optimized and positioned, which is still challenging and labor-intensive, often requiring advanced lithography techniques. Single NWs may also be fragile and prone to breakage, which may affect their long-term reliability and robustness in practical environments. Both architectures, while offering significant advantages in sensitivity and selectivity, face fabrication complexity, scalability, and integration challenges into electronic systems.

Random NW networks can offer a balanced approach that combines the sensitivity and functionality of nanowire-based sensors with simplified fabrication. Chemical vapor deposition (CVD), electrospinning, hydrothermal approaches, vapor–liquid–solid processes, and thermal oxidation are commonly used to prepare such NW structures [4,5,6,7,8,9,10,11]. In the following, we focus our interest on the thermal oxidation technique as it is a relatively simple and cost-effective method that provides good control of the thickness of the oxide layer by adjusting the oxidation time and temperature, allowing for fine-tuning of the nanowire properties. Moreover, it is compatible with a wide range of substrates and its process typically involves environmentally benign reactants (oxygen and water vapor). An additional reason for selecting thermal oxidation for NiO growth is the limited amount of research using this technique to prepare NiO nanowires, unlike ZnO, WO_3_, TiO_2_, and CuO materials [11,12,13,14,15,16]. N-type metal oxides are the most extensively studied semiconductors, while p-type metal oxides, which constitute only 10%, are less frequently explored [17]. We specify NiO as a promising p-type material known for its electrical properties, chemical stability, and catalytic activity, making it advantageous for applications such as gas sensing. However, it is challenging to synthesize high-quality NiO nanowires due to difficulties in controlling their morphology and crystallinity. NiO is also investigated herein because thermally oxidized NiO nanowires have not been previously reported for gas testing, which will be the challenge for this paper. Thermal oxidation of performed Ni nanowires is commonly found in the literature. Ren et al. [18] prepared NiO NWs by thermal oxidation from Ni NWs prepared by electrodeposition into anodic aluminum oxide (AAO) membranes, which are fabricated through a two-step anodization process and modified with phosphoric acid to form a through-pore template. After dissolving the template, the Ni nanowires were dispersed in ethanol and transferred onto a silicon nitride membrane for thermal oxidation. Xiang et al. also used thermal oxidation for Ni NWs synthesized via a chemical reduction-dropping method [19] by dissolving sodium hydroxide in ethylene glycol, followed by the addition of hydrazine hydrate as a reducing agent. The solution is heated to 80 °C in a magnetic field before nickel chloride hexahydrate solution is added dropwise, forming a black product [20]. A magnetic field is applied to collect the nanowires, which are then washed and dried at 60 °C for 12 h. The oxidation of Ni NWs at different temperatures shows significant changes in morphology. At 400 °C, the needle-like structures disappear, surface roughness decreases, and small cavities appear at the ends of the NWs. Increasing the temperature to 500–600 °C transforms the wire-like structures into tube-like structures, forming nanotubes (NTs) [19]. The oxidation temperature greatly affects the morphology of Ni nanowires. Additionally, previous experiments typically involve complex processes to form the nanowires, whereas thermal oxidation of a metallic nickel film is much simpler. Several studies have reported that oxidation of Ni film results in granular and porous structures [21,22,23,24,25]. In 2016, Zhu et al. [26] pioneered NiO nanowire growth via thermal oxidation of Ni foil, using an environment transmission electron microscope (ETEM) at 500 °C with controlled O_2_ pressure. Later studies in 2021 [27] and 2022 [28] oxidized 300 nm Ni NPs in an environment scanning electron microscope (ESEM) with oxygen/water vapor at 800 °C, achieving NiO nanorods. Koga and Hirasawa [29] used rapid oxidation up to 900 °C on laser-ablated Ni NPs to produce NiO nanorods. In contrast, our work simplifies this process by thermally oxidizing a sputtered Ni thin film at 800 °C under a steady O_2_ flow, without complex setups, offering a more scalable approach for NiO nanowire growth. The novelty and originality of this approach are explored in the present study for its potential application in gas sensing.

## 2. Materials and Methods

### 2.1. Samples

Nickel was layered through sputtering onto alumina substrates (Kyocera, Japan) with dimensions of 2 × 2 × 0.25 mm^3^ at room temperature for various durations. The thickness of the Ni thin film was modulated between 200 and 300 nm by adjusting the sputtering time. Subsequently, a nanolayer composed of a Au catalyst (or Pt catalyst) is applied onto the Ni layers prior to thermal oxidation to promote nanostructure growth. The oxidation process is conducted at 800 °C and 6 µbar for 4 h under an oxygen flow of 5 sccm. The samples were characterized by a Field Emission Gun Scanning Electron Microscope FEG-SEM MIRA3 from TESCAN, combined with energy dispersive X-ray spectroscopy, and a grazing incidence X-ray diffractometer which was Empyrean model, manufactured by PANalytical, Almelo, The Netherlands, working at 40 kV and 40 mA with a Cu-LFF source (λ = 1.54 Å).

### 2.2. Sensor Preparation

**Figure 1:**
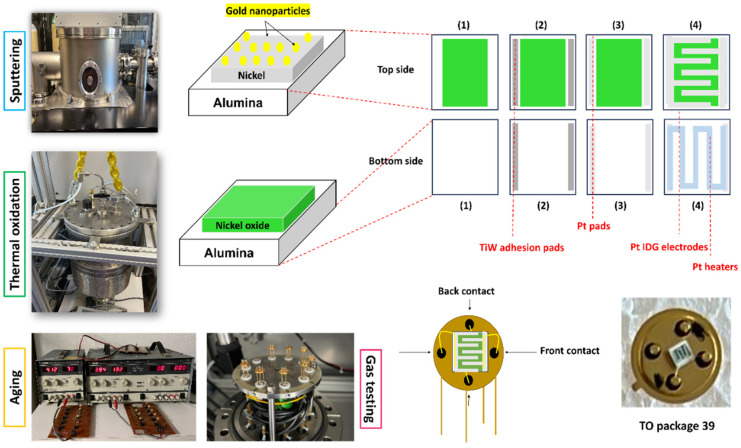
Sketch of NiO sensor preparation at the sensor laboratory.

Prior to the deposition of Pt electrical contacts, the samples were heated to 300 °C and subjected to deposition of Titanium–Tungsten (TiW) as adhesion pads of 1.8 × 0.3 mm^2^ (step (2) of Figure 1), onto which Pt pads with were deposited (step (3) of Figure 1), followed by deposition of interdigitated electrodes on the NiO sensing layer (top side of the alumina) using a multi-fingers mask (step (4) of Figure 1) with a width of 300 µm, a length of 1 mm, and a gap space of 200 µm between adjacent fingers. Regarding the bottom side of the alumina (step (1) of Figure 1), TiW and Pt pads (steps (2) and (3) of Figure 1) were also deposited, onto which Pt electrodes were patterned as heaters (step (4) of Figure 1). After the preparation of the electrical contacts, the samples were subjected to bonding with gold wires on a transistor outline (TO 39) package and to aging at 450 °C for two days to ensure the devices’ stabilization.

### 2.3. Gas-Testing Conditions

The experiment involved exposing the gas sensor to different gases using 200 sccm of total flow after thermal stabilization for 10 h and testing it across different operating temperatures under humid air (relative humidity: RH = 50% @ 20 °C). Based on the sensor’s selectivity, the device was exposed to suitable gas by varying concentrations of NO_2_, ranging from 0.5 ppm to 5 ppm at the optimum temperature, and studying its performance in a dry environment (RH = 0% @ 20 °C). The NO_2_ measurements were repeated to investigate the NiO sensors’ reliability and their long-term stability. The sensors were also subjected to exposure to different gases for selectivity tests. This was carried out by sequentially injecting each gas with a 30 min dose time and a 1.5 h recovery time in a testing chamber with a volume of 1 L.

Figure 1 summarizes the experimental procedures used for growing NiO film as a gas-sensing device.

## 3. Results and Discussion

### 3.1. Morphology and Structural Properties

**Figure 2:**
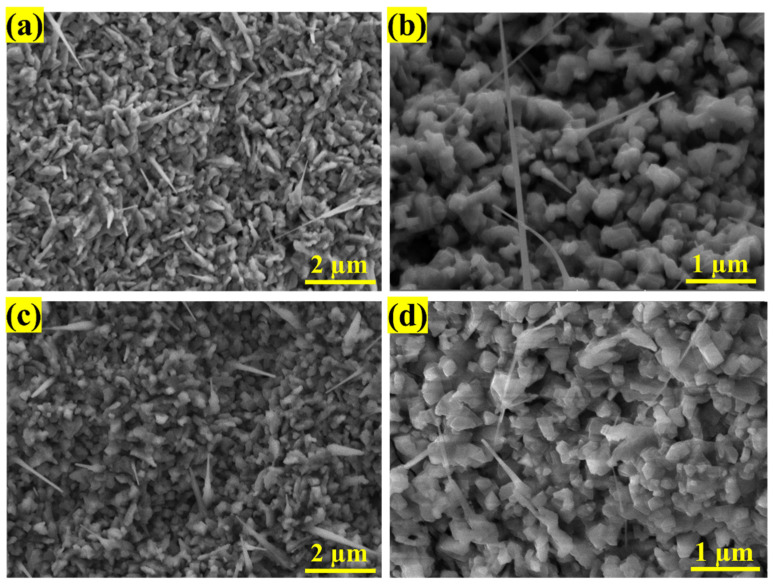
SEM images of (**a**,**b**) Pt-NiO and (**c**,**d**) Au-NiO structures oxidized at 800 °C.

Figure 2 shows the morphologies of NiO using two catalysts, Au and Pt. One can see the co-presence of granular and porous film with distribution of randomly oriented nanowires, which allows high accessibility and adsorption of gas species. The process begins with the deposition of a nickel (Ni) layer onto an alumina substrate, followed by the addition of catalyst nanoparticles such as gold (Au) or platinum (Pt) onto the nickel surface.

To explain the observed morphology of the NiO nanowires shown in Figure 2, we propose the following growth mechanism. The catalyst particles serve as active sites that initiate the oxidation of the nickel layer upon heating, promoting the formation of oxide nuclei, which act as seeds for the growth of nickel oxide nanowires. The growth of these nanowires is further driven by stress-induced grain boundary diffusion, where nickel ions migrate through the grain boundaries to react with oxygen atoms, facilitated by an oxygen concentration gradient [30].

**Figure 3:**
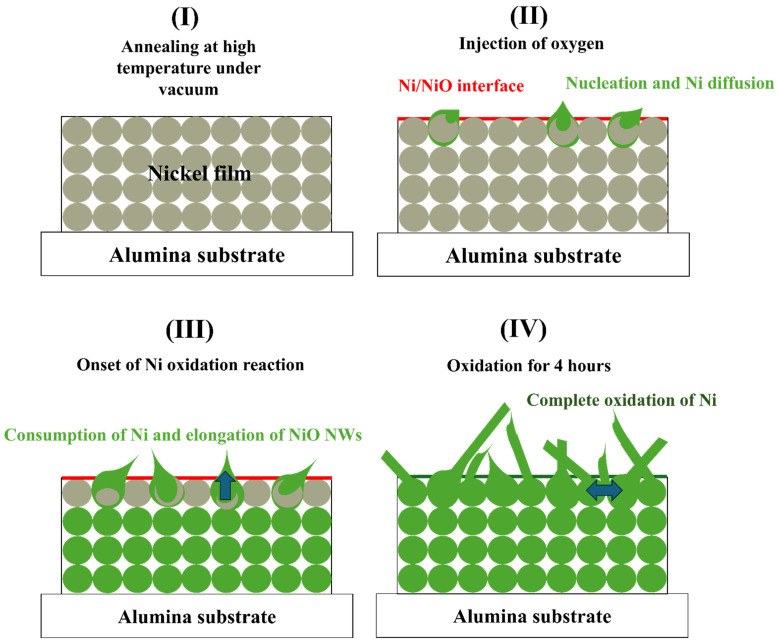
Representation of the Ni film oxidation process.

The formation of NiO nanowires from thermally oxidized Ni layers can be explained through a mechanism involving layer-by-layer oxidation at the Ni/NiO interface, similar to the process outlined in [28], where the authors used 300 nm of Ni NPs to be oxidized at 800 °C in an environment scanning electron microscope with oxygen/water vapor, resulting in NiO nanorods. In this mechanism, oxygen vacancies migrate along the interface plane, allowing Ni atoms to react with oxygen at kink sites, thereby driving growth along specific crystallographic directions. This process follows the Terrace Ledge Kink (TLK) model, where new NiO layers form progressively due to the migration of disconnections at the interface. As the Ni layer undergoes oxidation, Ni vacancies accumulate, leading to the gradual conversion of the Ni layer into NiO nanowires. The growth is primarily influenced by thermodynamic factors such as oxygen partial pressure and temperature, which promote oxygen migration and encourage the anisotropic formation of NiO. Over time, the Ni layer thins while NiO nanowires elongate, with growth rates governed by the balance between oxygen diffusion and interface step movement. This mechanism aligns with established crystal growth models, such as the Kossel–Stranski model, where atoms preferentially attach to energetically favorable sites, adapting to the unique conditions of Ni thermal oxidation. A detailed schematic representation of the Ni film oxidation process is provided in Figure 3.

As noted in reference [31], the optimization of small grains and large pores is essential for enhancing both the transduction process and the efficiency of gas diffusion within the entire volume of the sensing material. However, in conventional thick films, a common challenge arises where small particles tend to be associated with small pores, possibly leading to counterproductive outcomes. To address this challenge, reducing the film thickness and integrating nanowires into these granular and porous structures can be effective by improving gas adsorption on the sensing material and increasing the specific surface area [32]. This approach forms a NiO network that interconnects Pt interdigitated electrodes, facilitating efficient interaction with gas species whose sensing capabilities will be investigated in Section 3.2.

**Figure 4:**
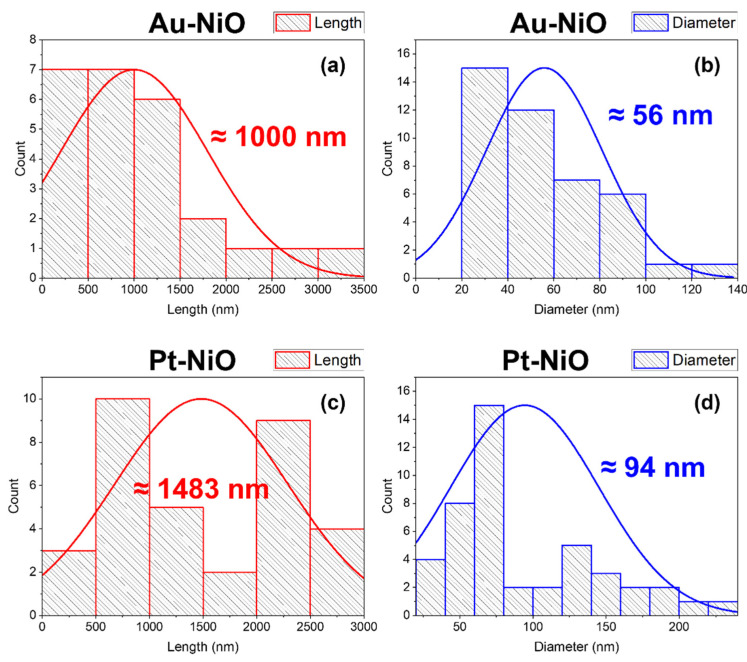
(**a**,**c**) Length distribution and (**b**,**d**) diameter distribution of nanowires for Au-NiO and Pt-NiO with a thickness of 200 nm.

To further characterize the morphology of the NiO nanowires, we analyzed their size distribution based on SEM images. The statistical analysis revealed that the average nanowire length is approximately 1000 nm for Au-NiO and 1483 nm for Pt-NiO, while the average diameter is 56 nm for Au-NiO and 94 nm for Pt-NiO. These differences indicate that the choice of catalyst significantly influences the growth of NiO nanowires. The Pt-catalyzed nanowires exhibit a greater length and diameter compared to their Au-catalyzed counterparts, suggesting a more efficient catalytic effect in promoting nanowire elongation. This variation in morphology can be attributed to the distinct catalytic activity of Au and Pt, which affects the nucleation and diffusion kinetics of Ni species during the thermal oxidation process. The size distribution histograms of NiO nanowires for both catalysts are presented in Figure 4, providing a comprehensive view of the structural differences.

**Figure 5:**
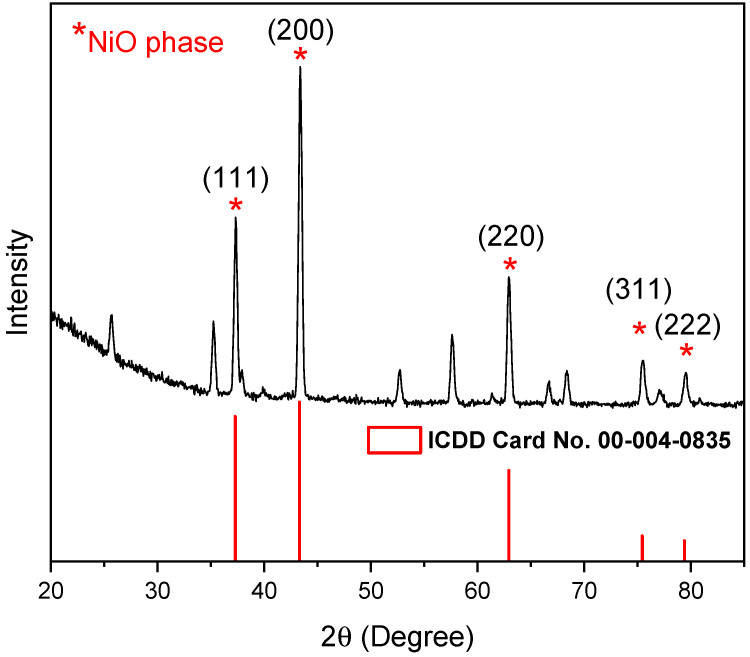
GIXRD patterns of Pt-NiO film (450 nm) oxidized at 800 °C for 4 h.

Figure 5 presents the grazing incident X-ray diffraction (GIXRD) patterns of a NiO sample oxidized at 800 °C.

The NiO phase is identified according to ICDD card 00-004-0835. The most significant Bragg’s reflection peaks correspond to crystal planes (111), (200), (220), (311), and (222) and are, respectively, located at 2θ values of 37.32°, 43.38°, 62.92°, 75.47°, and 79.52°, with preferred orientation along (200). The other peaks are related to the alumina substrate according to ICDD cards No. 01-073-1512 and 00-001-1305. The prepared NiO structure is polycrystalline and is depicted to be a cubic single phase of bunsenite NiO with space group Fm3m: 225.

The crystallinity degree of nickel oxide (NiO) was determined based on the analysis of the X-ray diffraction (XRD) patterns presented in Figure 5. This parameter quantifies the proportion of the crystalline phase within the material, providing insights into its structural characteristics and potential impact on its electronic and gas-sensing properties. The crystallinity index was calculated by integrating the area of the crystalline peaks relative to the total area of the diffraction pattern, which includes both crystalline and amorphous contributions. The obtained value of 31.22% suggests a significant degree of structural disorder in the NiO film. This partial amorphous nature may be attributed, in part, to the influence of the alumina substrate, which can contribute to an overall reduction in crystallinity due to lattice mismatch, limited grain growth, or induced defects.

In addition to crystallinity, the XRD data also allow for the estimation of strain and stress effects within the NiO film. The broadening of diffraction peaks can be associated with microstructural strain, which arises from factors such as defects, dislocations, and lattice distortions.

The crystallite size D, dislocation density δ, and lattice strain ε for NiO were determined using the following equations [33]:

(1) $$D_{\mathrm{cristallite}}=\frac{M\;\lambda }{\beta \;\cos\theta }$$

where M = 0.9 is the dimensionless shape factor, λ = 1.54 Å is the wavelength of the incident X-ray, β is the full width at half maximum (FWHM) extracted from the XRD pattern, and θ is the Bragg diffraction angle identified from the XRD spectrum.

The dislocation density δ and lattice strain ε are calculated as:

(2) $$\delta =\frac{1}{D^{2}}$$

(3) $$\frac{\beta \;\cos\theta }{4}$$

Additionally, the interplanar spacing d is determined using Bragg’s law:

(4) $$d=\frac{n\;\lambda }{2\;\sin\theta }$$

where n represents the reflection order.

All the parameters have been estimated and are presented in Table 1.

**Figure 6:**
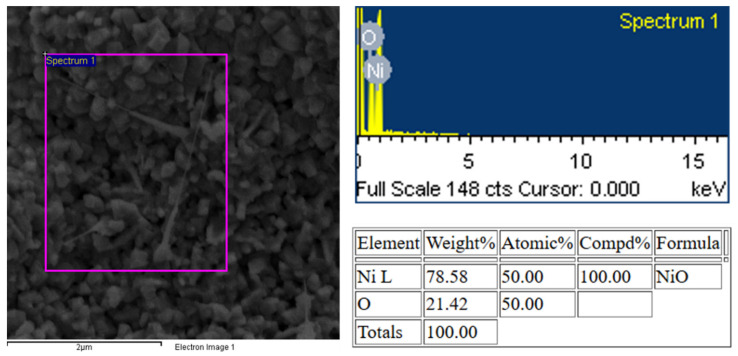
EDX analysis of the Pt-NiO (200 nm) sample.

The analysis of the NiO structure reveals that the (200) crystal plane emerges as the dominant orientation. This preferred orientation suggests that the synthesis conditions favored the growth of NiO along this specific crystallographic plane. The prominence of the (200) plane further implies that the NiO structure was formed with a stoichiometric composition, meaning the ratio of nickel to oxygen atoms aligns with the ideal 1:1 NiO formulation [34]. The EDX analysis shown in Figure 6 proves that the primary material in the sample is NiO, with a stoichiometric Ni atomic ratio of 1:1, as expected for pure nickel oxide.

Achieving such a stoichiometric balance is important, as it confirms that the structures have a well-defined and stable composition, free from significant defects like oxygen vacancies or excess nickel atoms. The formation of NiO with a dominant (200) orientation also suggests that the nanostructures may exhibit consistent surface properties, which can influence their stability, reactivity, and overall performance in applications.

### 3.2. Gas-Sensing Performance

#### 3.2.1. Gas Response at Different Operating Temperatures

**Figure 7:**
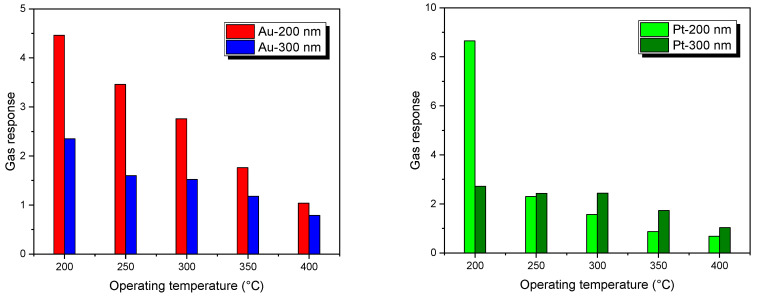
Dependency of NO_2_ response on an operating temperature of RH = 50% @ 20 °C for different NiO sensors.

Figure 7 shows the NiO response toward NO_2_ at different thicknesses using Au and Pt catalysts in a temperature range of 200–400 °C and in a wet air environment (relative humidity RH = 50%).

The optimal operating temperature of 200 °C for NiO sensors toward NO_2_ with varying thicknesses and catalysts is likely due to a combination of optimal surface reaction kinetics, enhanced catalyst activity, and favorable charge carrier mobility. Penza et al. reported that the gas sensitivity of multi-walled carbon nanotube (MWCNT)-based chemoresistors can be tailored by the type of surface catalyst. Specifically, platinum and gold catalysts enhance the sensitivity to NO_2_, respectively, due to metal-induced gap states [35]. In our NiO gas testing, platinum shows a better sensing response compared to gold that can be assigned to higher catalytic activity and more active sites for gas adsorption and reaction with NiO surfaces and NO_2_. Pt may modify the electronic properties of NiO more significantly than Au, enhancing the overall sensitivity of the sensor [36,37,38].

At 200 °C, the morphology of the NiO layer might be such that it provides optimal surface area and porosity for gas diffusion and interaction, leading to improved sensitivity. Also, the energy provided at 200 °C to the sensor surface is sufficient to activate the catalytic reaction between NO_2_ and the surface of NiO. This temperature likely offers the best balance between adsorption and desorption rates of NO_2_ on the NiO surface, leading to maximum response. Catalysts are often used to lower the activation energy of a chemical reaction. At 200 °C, the catalysts on the NiO surface reach an optimal activation state, facilitating efficient interaction with NO_2_ molecules. This can enhance the sensor’s response to NO_2_ by increasing the reaction rate on the surface, noting that the Pt catalyst shows a higher response compared to the Au catalyst. Thinner films (200 nm) exhibit a more pronounced response compared to thicker layers. Another explanation was reported in the literature [39] in a study of the gas concentration profile as a function of sensing layer thickness based on Knudsen diffusion and Langmuir adsorption models. It was proved that by increasing the thickness of the film, the amount of gas species adsorbed on the sensing layer decreases drastically. This behavior weakens the surface interactions with gas molecules and thus decreases the metal oxide sensor’s responsiveness. Experimental works have also found similar results for NiO sensors [40]. Temperature significantly influences the operation of gas sensors based on metal oxides. The response of NiO gas sensors is highly dependent on temperature variations, as shown in Figure 7. This dependency arises due to several factors [41]. Firstly, the adsorption and desorption rates of gas molecules onto the surface of the metal oxide are temperature dependent. At higher temperatures, the adsorption of gas molecules onto the surface of the MOX material increases, leading to a more pronounced response to the target gas. Secondly, the activation energy required for the chemical reaction between the gas molecules and the metal oxide surface also varies with temperature. At the optimal temperature (200 °C for our sensors), the activation energy is reached, resulting in efficient gas detection. Moreover, the temperature affects the conductivity of the NiO material, influencing the electrical signal generated by the sensor in response to NO_2_ exposure.

In the following, we will focus on Au-NiO and Pt-NiO sensors with a thickness of 200 nm, as they exhibit the highest response compared to other samples.

#### 3.2.2. Dynamic Response to NO_2_

**Figure 8:**
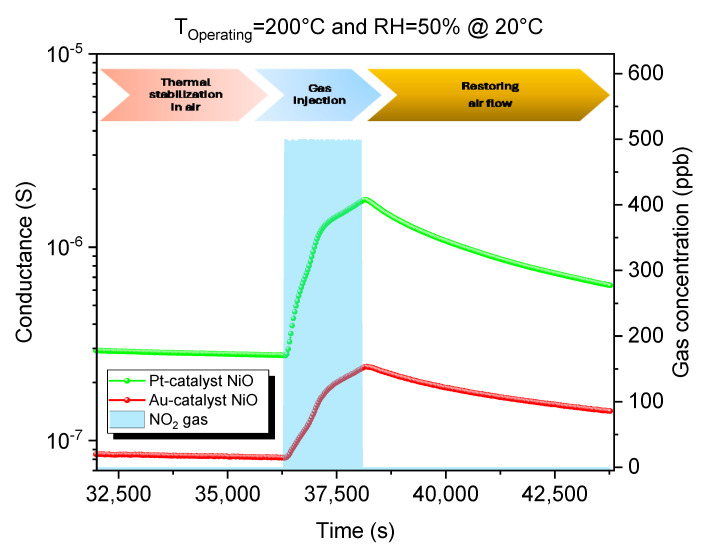
Dynamic response curves with respect to 0.5 ppm NO_2_ at 200 °C and RH = 50% @ 20 °C for Au catalyst NiO sensors.

The operational principle of NiO in our gas chamber involves altering the material’s electrical conductance due to variations in free hole density resulting from physisorption, chemisorption, and catalytic reactions between the NiO material’s surface and the target gas. The dynamic curves of Pt catalyst and Au catalyst NiO sensors when exposed to oxidizing gas reflect the p-type characteristic of NiO films, as Figure 8 illustrates.

When exposed to NO_2_, electrons from the valence band are believed to be trapped at the surface, forming ${\mathrm{NO}}_{2}^{-}$ species and increasing the hole concentration near the surface, according to reaction (5) [42]:

(5) $${\mathrm{NO}}_{2}\left(g\right)\to \;{\mathrm{NO}}_{2}^{-}\left(\mathrm{ads}\right)+h^{+}$$

Since holes are the majority charge carriers, this significantly boosts the electrical conductivity of the NiO material. ${\mathrm{NO}}_{2}^{-}$ and molecular/atomic oxygen are two competing species on the NiO adsorption sites, which results in longer recovery transients, as shown in Figure 8, due to the difference in electron affinity (2.3 eV for NO_2_ and 0.4 eV for molecular oxygen) [43]. Nickel vacancies in NiO significantly enhance its gas-sensing properties with respect to NO_2_ by creating additional active sites for adsorption, leading to increased sensitivity [44]. These vacancies, often positively charged, facilitate stronger interactions with the electronegative NO_2_ molecules, resulting in higher adsorption energy and a more pronounced gas response. Furthermore, the vacancies enable efficient electron transfer between the NO_2_ molecules and the NiO surface, altering the material’s conductivity, which is crucial for detecting gas presence. Additionally, interaction with NO_2_ can ionize nearby neutral vacancies, further increasing the number of adsorption sites and amplifying the sensor’s effectiveness.

#### 3.2.3. Mechanism of NO_2_ Sensing

The gas-sensing mechanism of semiconducting NiO gas sensors relies on an alteration in the electrical conductance of the sensing material, where the sensing response will be measured as $G=\frac{G_{\mathrm{gas}}-G_{\mathrm{air}}}{G_{\mathrm{air}}}$ for oxidizing gas and $G=\frac{G_{\mathrm{air}}-G_{\mathrm{gas}}}{G_{\mathrm{gas}}}$ for reducing gas. $G_{\mathrm{gas}}$ and $G_{\mathrm{air}}\;$denote the stable conductance of the sensor in a gas atmosphere and the initial conductance of the sensor in an air atmosphere, respectively.

Upon exposure to air, oxygen molecules adhere to the NiO surface, initiating a process of electron capture from the semiconductor’s conduction band. This leads to the ionosorption of oxygen in molecular form and atomic forms. The presence of these oxygen species depends on the operating temperature.

**Figure 9:**
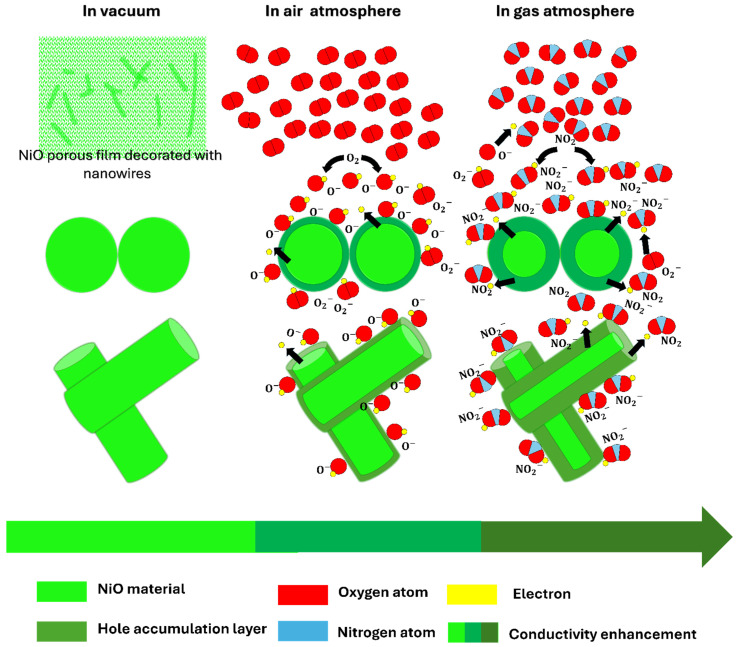
Schematic description of gas-sensing mechanisms.

Upon NO_2_ exposure, electron capture further increases due to the high electronegativity of the oxidizing molecules, resulting in an increase in the conductance of NiO (rise in hole concentration), characteristic of p-type semiconductors, as presented in Figure 9.

The interaction between NiO and NO_2_ involves complex surface chemistry, with the type of oxygen species and their reactivity being strongly temperature-dependent.

Low temperatures (<180 °C): Oxygen molecules are weakly adsorbed on the NiO surface and ionized to form $O_{2}^{-}$. However, due to insufficient thermal energy, the adsorption and ionization processes are slow, leading to weaker NO_2_ interaction, a delayed sensor response, and incomplete recovery.

Moderate temperatures (180–400 °C): Molecular oxygen dissociates into atomic oxygen, forming $O^{-}$, which is more reactive than $O_{2}^{-}$. The enhanced surface activity increases NO_2_ adsorption, leading to a significant rise in hole concentration and, consequently, higher sensor sensitivity. The improved interaction between NO_2_ and $O^{-}$ species results in faster and more efficient sensing.

High temperatures (>400 °C): Oxygen species on the NiO surface further ionize into $O^{2-}$, increasing surface reactivity. However, the stronger bonds of $O^{2-}$ may reduce its reactivity toward NO_2_ compared to $O^{-}$, leading to fewer available active sites. At these temperatures, desorption of reaction products is more efficient, allowing for complete and rapid sensor recovery.

Reactions (6)–(9) describe the oxygen adsorption and surface interaction in different temperature ranges in an air atmosphere [45,46].

(6) $$O_{2}^{\left(\mathrm{gas}\right)}\to O_{2}^{\left(\mathrm{ads}\right)}$$

(7) $$O_{2}^{\left(\mathrm{ads}\right)}+e^{-}\to O_{2}^{-}$$

(8) $$O_{2}^{-}+e^{-}\to 2O^{-}$$

(9) $$O^{-}+e^{-}\to O^{2-}$$

After exposure to NO_2_, the NiO surface reacts with the oxidizing species, according to reactions (10)–(13) [45,47].

(10) $${\mathrm{NO}}_{2}^{\left(\mathrm{gas}\right)}\to {\mathrm{NO}}_{2}^{\left(\mathrm{ads}\right)}$$

(11) $${\mathrm{NO}}_{2}^{\left(\mathrm{ads}\right)}+e^{-}\to {\mathrm{NO}}_{2}^{-}$$

(12) $${\mathrm{NO}}_{2}^{\left(\mathrm{ads}\right)}+O_{2}^{-}+2e^{-}\to {\mathrm{NO}}_{2}^{-}+2O^{-}$$

(13) $${\mathrm{NO}}_{2}^{-}+2O^{-}+e^{-}\to {\mathrm{NO}}_{2}^{\left(\mathrm{gas}\right)}+2O^{2-}$$

The physisorption of NO_2_ molecules leads to new acceptor levels, deeper than oxygen levels ($O^{-}$ and $O_{2}^{-}$), which will be chemisorbed (${\mathrm{NO}}_{2}^{-}$) and result in band bending at the NiO surface due to the difference in electronegativity. This implies a conductivity increase in the material, as schematized in Figure 9.

Table 2 provides a comprehensive summary of NO_2_ sensors based on metal oxides synthesized through thermal oxidation, highlighting their key performance metrics.

Table 3 presents a review of the performance of NiO sensors fabricated using various synthesis methods, comparing their sensitivity, operating conditions, and gas-sensing properties.

The NiO sensor developed in this work through thermal oxidation demonstrates a high response (G = 8.65) to 5 ppm of NO_2_ at 200 °C, outperforming many reported metal oxide sensors and NiO-based sensors synthesized using different methods. Compared to other NO_2_ sensors in Table 2, our sensor exhibits a significantly higher response at a lower concentration. For instance, ZnO nanorods [51] show a weak response of R = 1.01 to 100 ppm of NO_2_, while ZnSe/ZnO mesoporous structures [52] achieve a response below 7 for 5 ppm at 200 °C, which is still lower than our sensor.

When compared to NiO sensors synthesized through different methods in Table 3, our sensor maintains a strong advantage. For example, hydrothermally grown NiO nanosheets [57] show an extremely low response (G = 0.39 to 15 ppm of NO_2_ at room temperature), while NiO nanoparticles prepared via co-precipitation [58] exhibit a response in the range 1.75–2.25 at 180 °C for 5 ppm of NO_2_, both of which are significantly weaker than our sensor. Even compared to NiO sensors prepared by thermal oxidation, such as the granular film for H_2_S detection [54] with R′ = 2 at room temperature, our sensor demonstrates a much stronger response, albeit at a slightly higher operating temperature.

In comparison to previous studies on NiO nanowire growth through oxidation, our approach offers greater simplicity and scalability. While studies by Zhu et al. [26], Koga and Hirasawa [29], and others [27,28] rely on advanced techniques such as ESEM, ETEM, and specific starting materials like Ni nanoparticles (NPs), our method utilizes a Ni-sputtered thin film, eliminating the need for complex nanoparticle preparation or in situ monitoring. Moreover, previous studies have not explored the gas-sensing capabilities of NiO nanorods and nanowires, preventing direct performance comparison with thermally oxidized NiO nanowires. However, NiO nanowires synthesized via other methods have shown comparable or even superior NO_2_-sensing responses. For instance, reference [62] reports that long, thin, and densely packed NiO nanowires (20–60 nm in diameter) exhibited a response of 6 to 1 ppm of NO_2_ at 200 °C, with an even higher response expected at 5 ppm, surpassing that of Pt-NiO sensors with larger nanowire diameters (~94 nm). Additionally, NiO nanowires grown via the vapor–liquid–solid process demonstrated an impressive response of 54.8 to 1 ppm of NO_2_ at 200 °C, attributed to their high density and smaller diameters (10–50 nm). These findings highlight the critical role of nanowire morphology in enhancing gas-sensing performance.

**Figure 10:**
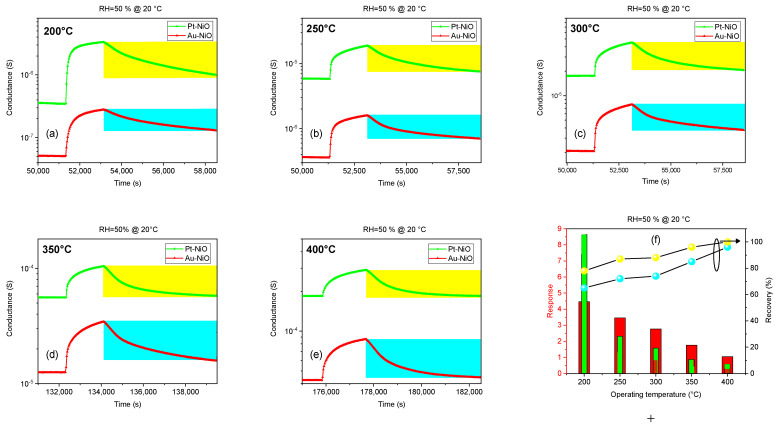
Dynamic response–recovery curves of Au catalyst and Pt catalyst NiO sensors with respect to 5 ppm of NO_2_ at different working temperatures: (**a**) 200 °C, (**b**) 250 °C, (**c**) 300 °C, (**d**) 350 °C, and (**e**) 400 °C, with (**f**) temperature dependency in terms of their gas response and recovery rate at 50% of relative humidity. The yellow and blue areas indicate the recovery time for Pt-NiO and Au-NiO sensors, respectively. The green and red bars represent the response intensity of Pt-NiO and Au-NiO sensors, respectively. The yellow and blue lines correspond to the recovery characteristics of the Pt-NiO and Au-NiO sensors, respectively.

**Figure 11:**
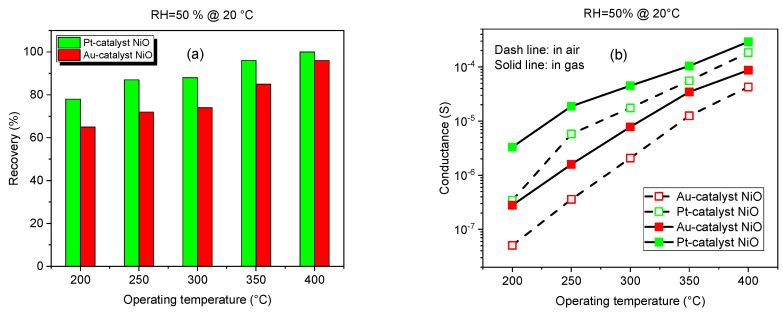
(**a**) Histogram representation of recovery versus operating temperature at 50% of relative humidity, and (**b**) temperature-dependent baseline conductance for Au- and Pt-NiO sensors.

The operating temperature plays a crucial role in the recovery of our NiO sensors after exposure to NO_2_, as shown in Figure 10 and Figure 11. Recovery refers to the sensor’s ability to return to its baseline or initial state after interacting with a specific gas concentration. One can mention the increase in recovery by varying the temperature from 200 °C to 400 °C. Increasing the operating temperature can accelerate the desorption of gas molecules, specifically NO_2_ species that have high adsorption from the sensor’s surface, leading to a faster recovery time. Higher temperatures provide the necessary energy to break the bonds between the gas molecules and the nickel oxide surface, facilitating a quicker return to baseline readings.

Before exposure to NO_2_, at lower temperatures, the conductivity of the NiO sensor is relatively low [63]. As the temperature increases, the conductivity of NiO typically increases due to the high concentration and enhanced mobility of free charge carriers within the material. This temperature-dependent conductivity is crucial for the sensor’s operation, as it affects baseline conductance and response characteristics, as mentioned in Figure 11b.

The adsorption of NO_2_ molecules onto the NiO surface can lead to the transfer of charge between the gas molecules and the Ni vacancies. This charge transfer process results in altering the conductivity of the NiO sensor via following the reactions (14)–(16) [41].

(14) $${\mathrm{NO}}_{2}^{\left(\mathrm{gas}\right)}\to {\mathrm{NO}}_{2}^{\left(\mathrm{ads}\right)}$$

(15) $${\mathrm{NO}}_{2}^{\left(\mathrm{ads}\right)}+V_{Ni}^{\prime \prime }\to {\mathrm{NO}}_{2}^{-\left(\mathrm{ads}\right)}+V_{Ni}^{\prime }$$

(16) $$V_{Ni}^{\prime }\to V_{Ni}^{\prime \prime }+h^{+}$$

At an optimum temperature, the sensor exhibits the highest response to NO_2_, as an equilibrium between the adsorption and desorption kinetics of NO_2_ molecules is achieved. It is worth noting that high temperatures can affect the thermal stability of Ni vacancies. If the temperature exceeds a certain threshold, it could lead to the recombination of vacancies with other defects or atoms, reducing the number of available active sites and potentially decreasing the sensor’s responsiveness, as described in Figure 10f.

The desorption of NO_2_ molecules from the NiO surface occurs, leading to a decrease in the concentration of surface adsorption sites and a restoration of the sensor’s baseline conductance. The desorption rate is also temperature dependent: it increases with higher temperatures, facilitating faster desorption kinetics. At lower temperatures, a higher proportion of NO_2_ adsorption may be irreversible, meaning that some NO_2_ molecules form strong and potentially permanent bonds with the sensor surface. The recovery time after exposure to NO_2_ was notably lengthy for Au-NiO and Pt-NiO sensors, taking hours, resulting in subsequent gas exposures occurring before the sensors had fully recovered. This prolonged recovery time for NO_2_ is a common observation in terms of NiO-based sensors.

At higher temperatures, the adsorption process is more reversible, allowing the sensor to fully recover after NO_2_ exposure. Those implications are proved in Figure 11a,b by describing the recovery rate and the baseline conductance at different operating temperatures, showing higher recovery and higher conductance by increasing the temperature from 200 °C to 400 °C.

**Figure 12:**
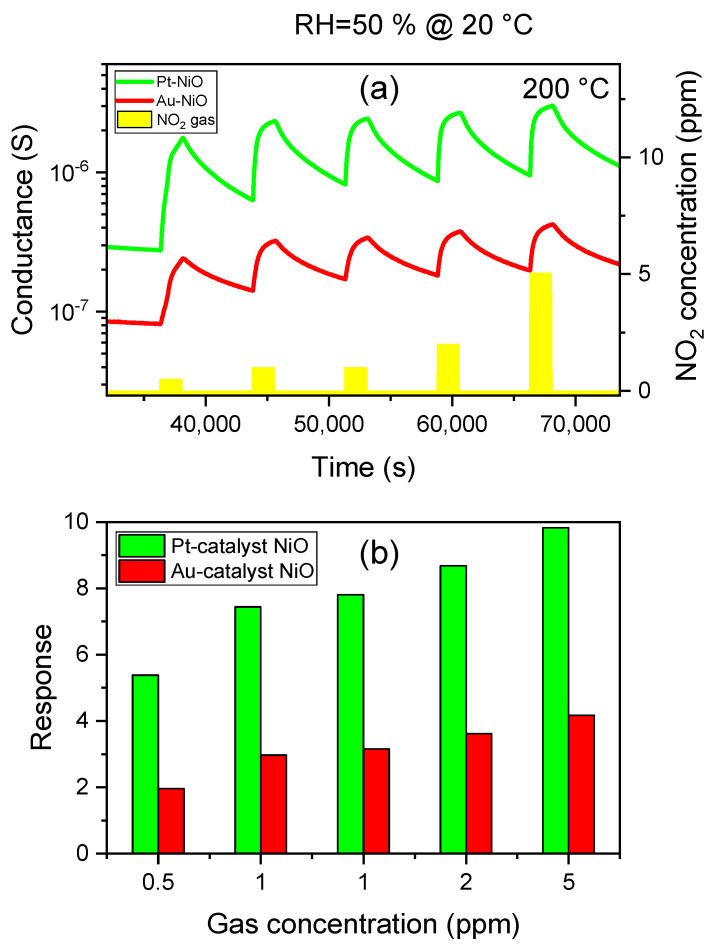
(**a**) Dynamic response–recovery curves of Au-NiO and Pt-NiO sensors under exposure to NO_2_ at different concentrations at 200 °C and 50% of relative humidity. (**b**) is the histogram representation of NO_2_ response for both sensors.

The NiO sensors demonstrated good reproducibility after undergoing two cyclic measurements (1 ppm) at the optimum condition (See Figure 12). During air injection into the gas chamber, the sensors cannot return to their initial baseline, as seen in Figure 12a. This behavior is due to the high binding energy of NO_2_ molecules, which can be addressed by thermal activation, as shown in Figure 10, optical excitation, and formation of heterojunctions, which provide fresh active sites and enhance adsorption–desorption rates [64].

#### 3.2.4. Moisture Effect

**Figure 13:**
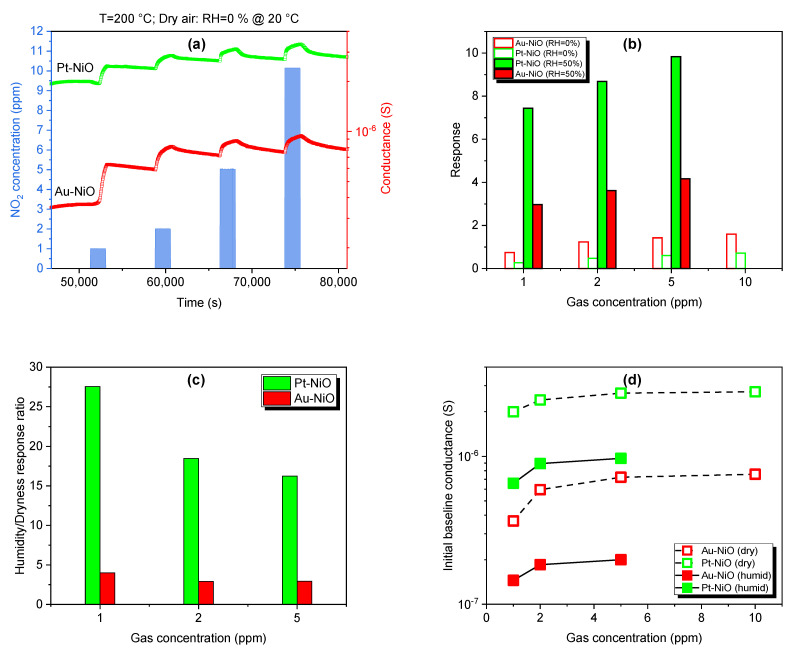
(**a**) Dynamic response–recovery curves of Au catalyst and Pt catalyst NiO sensors under exposure to NO_2_ at different concentrations at 200 °C in a dry atmosphere. (**b**,**c**) are the histogram representations of NO_2_ response in dry and humid environments and the humidity-to-dryness response ratio for both sensors. (**d**) represents the initial baseline conductance before exposure to gas for the sensors in dry and humid atmospheres.

Cross-interference with humidity significantly limits the reliability and accuracy of gas detection in metal oxide gas sensors. In humid environments, water molecules readily deactivate the active sites on the surface of metal oxides that are crucial for gas adsorption, hence limiting their gas response. P-type semiconductors are known for their low humidity dependency. However, NiO sensors show better sensing performance in humid air, unlike dry air. In Figure 13, we exhibit the dynamic curves in dry air under exposure to NO_2_ and the response toward various gas concentrations in dry and wet environments to study the effect of humidity on the sensing performance of NiO samples. One can see that humid environments show a higher response compared to dry environments for Au- and Pt-NiO sensors. Similar observations are made for silicon dioxide as a p-type material, which is more tolerant to moisture under exposure to NO_2_ [65]: NO_2_ has a stronger interaction with the surface compared to water and oxygen molecules, making it the most favorable reactant. The gas response of the SiO_2_ sensor to 0.2 ppm of NO_2_ is enhanced in humid air (RH = 50%), with an average increase of 30% compared to the response in dry air (RH = 0%). A higher increase in NiO responsiveness is recorded for Pt-NiO (×2700%) and Au-NiO (×400%) sensors when changing the environment from dry to humid under exposure to 1 ppm of NO_2_ (See Figure 13c).

**Figure 14:**
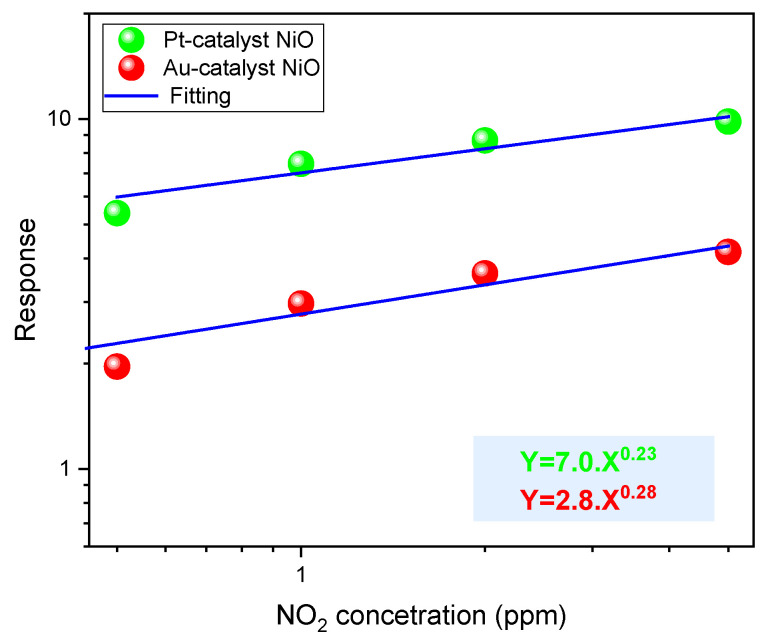
Calibration of NiO sensors with a thickness of 200 nm in optimum conditions.

Following the conventional power law for gas response $G=\alpha .c^{n}$ applicable to metal oxides for gas-sensing applications and the 100% method (to achieve a minimum response of 1), we calculate the limit of detections LOD = 26 ppb and LOD = 0.2 ppb for Au-NiO and Pt-NiO sensors, respectively, based on the calibration presented in Figure 14. The parameters α, c, and n are indicative of the sensitivity parameter (ppm^−1^), the gas concentration of nitrogen dioxide (ppm), and the surface reaction kinetics between NO_2_ molecules and the predominant oxygen species at 200 °C.

Breathing high levels of NO_2_ can irritate the respiratory system and worsen conditions like asthma. The Occupational Safety and Health Administration (OSHA) provides permissible exposure limits (PELs) for NO_2_ in the workplace. According to OSHA, the PEL for NO_2_ is 5 ppm as a ceiling limit, which should not be exceeded during any part of the working day. Another organization, the National Institute for Occupational Safety and Health (NIOSH), recommends 1 ppm and 40 ppb as short-term exposure limits (STPELs) at 1 h and 24 h, respectively. Our sensors have an exceptionally low limit of detection, capable of identifying NO_2_ concentrations as low as 0.2 to 26 ppb. This sensitivity allows the NiO sensors to detect NO_2_ at levels significantly below the short-term exposure limits set by both OSHA and NIOSH. This ensures high safety and effective monitoring, providing protection well beyond the recommended exposure limits for nitric acid gas. However, at optimal conditions, NiO sensors show incomplete recovery due to the high binding energy of NO_2_ molecules on the sensor surface. At a higher operating temperature of 400 °C, the sensors achieve full recovery, yet the response is reduced compared to 200 °C. Specifically, for Pt-NiO sensors, the response at 200 °C is 12.72 times that at 400 °C, and for Au-NiO, it is 4.28 times greater at 200 °C than at 400 °C. Thus, while elevated temperatures can improve recovery, they reduce sensor response, highlighting a trade-off between sensitivity and recovery efficiency.

**Figure 15:**
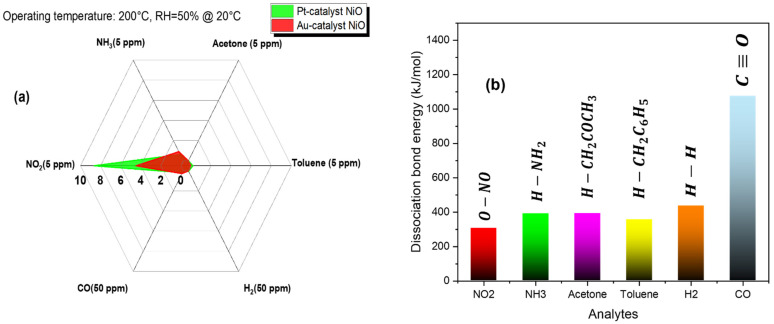
(**a**) Gas selectivity to nitrogen dioxide, ammonia, acetone, toluene, hydrogen, and carbon monoxide at the optimum operating temperature. (**b**) dissociation bond energies of each tested gas [47,66,67].

Additional critical measurements, such as selectivity and stability, are conducted to validate the overall sensing performance of NiO sensors. In Figure 15, we measured sensitivity and selectivity under identical operating conditions toward various analytes: NO_2_, NH_3_, acetone, toluene, H_2_, and CO.

A closer look at the bar chart shown in Figure 15b can justify the response of the sensors toward reducing and oxidizing gases: high NO_2_ selectivity is associated with the lowest dissociation-bond energy for O−NO among the other gases’ energies. The high electronegativity of NO_2_ molecules also explains the highest response compared to the response relative to the reducing gases. Wang et al., using DFT calculations, determined the adsorption energies of NO_2_ and NH_3_ on the NiO surface [68]. They found that the interaction between NO_2_ molecules and the NiO surface was the strongest. For instance, when comparing NO_2_ to NH_3_ gas, we find that the oxidizing gas has lower adsorption energy ($E_{\mathrm{ads}}^{{\mathrm{NO}}_{2}}=-0.310\;\mathrm{eV}/\mathrm{molecule}$ vs. $E_{\mathrm{ads}}^{{\mathrm{NH}}_{3}}=-0.101\;\mathrm{eV}/\mathrm{molecule}$ [68]), which implies stronger binding to the surface, higher adsorption, and better sensing response. Complementary in situ DRIFT studies by Zhang et al. revealed that a higher number of nickel vacancies in NiO nanostructures enhanced their sensitivity to NO_2_ [69]. They realized that the strong interaction between doubly charged nickel vacancies and NO_2_ species through electron transfer promoted the ionization of neutral nickel vacancies, thereby creating more adsorption sites for the analyte and enhancing the sensing property. The adsorption energy also accounts for the gas response to 50 ppm of CO and hydrogen gases, where the CO response (18.40%) is higher than that of H_2_ (4.75%). This difference can be attributed to the lower adsorption energy of CO on the NiO surface, as calculated by Nie et al., with $E_{\mathrm{ads}}^{\mathrm{CO}}=-1.496\;\mathrm{eV}$ compared to $E_{\mathrm{ads}}^{H_{2}}=-0.146\;\mathrm{eV}$ [70].

**Figure 16:**
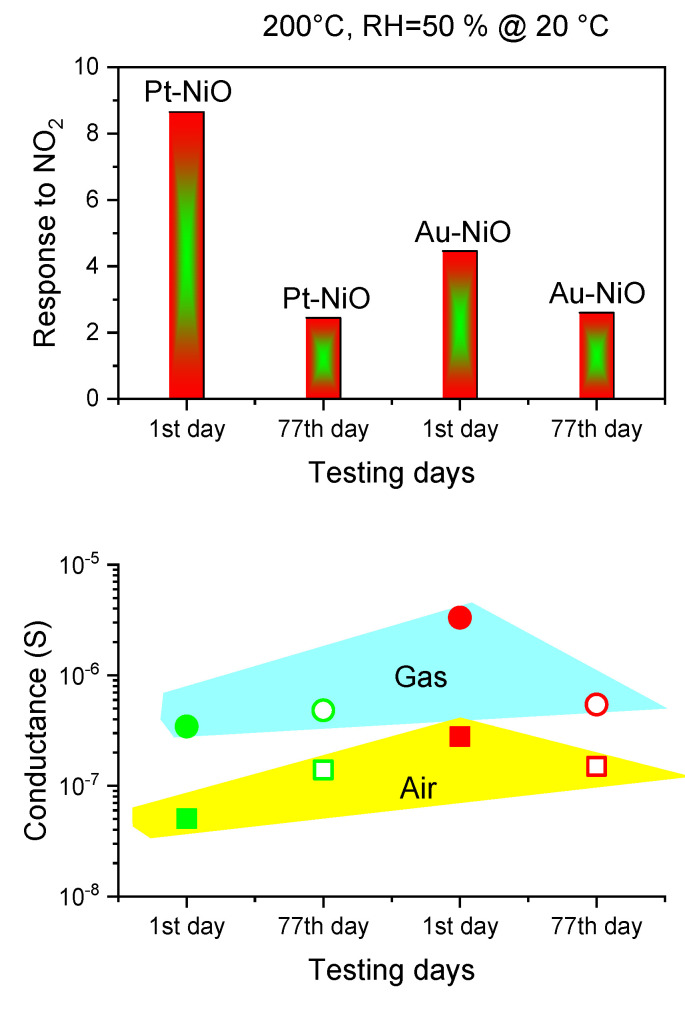
Long-term stability of NiO sensors: response to NO_2_ and initial conductance in air and gas over 77 days. The green and red shapes indicate the conductance of Pt-NiO and Au-NiO sensors, respectively.

Regarding the long-term stability protocol, the measurements were conducted over a period of 77 days, as shown in Figure 16. These were not continuous but rather taken at selected intervals to assess sensor stability over time. The response decrease toward NO_2_ by three orders for Pt-NiO sensors can be addressed in terms of the possible remaining gas on the active sites of NiO after tens of cyclic measurements in succession, noting that the initial conductance in air, described in Figure 16, increases on the 77th day, compared to the first day (high surface conductivity may be explained by the presence of NO_2_ molecules on the surface as they have strong affinity and adsorption energy).

Wang et al. demonstrated, via molecular simulation, that NO_2_ has strong interaction on NiO surfaces, leading to the formation of additional adsorption monolayers of the oxidizing gas, which saturate the reactive sites and make it challenging to completely remove the gas species from the surface, resulting in a lower sensing response [68]. Another factor to consider when working with p-type materials is the Mars–van Krevelen mechanism [46]: the reactivity of lattice oxygen can significantly impact the sensing performance of metal oxides over time. The surface lattice oxygen is more prone to losing electrons to bulk oxygen, thereby becoming reactive and participating in surface reactions. Over time, this loss of lattice oxygen could explain the observed decline in the performance of p-type materials, such as the drastic decrease in the sensitivity of NiO sensors over 77 days.

## 4. Conclusions

In conclusion, this study has successfully demonstrated a straightforward and cost-effective method for fabricating porous NiO films decorated with nanowires, achieved through sputtering followed by thermal oxidation of the Ni layer with two thicknesses using different catalysis. The structural analysis confirmed the presence of a single NiO phase and the formation of nanowires within the porous matrix. Our investigation into the gas-sensing properties of these films, particularly towards NO_2_, highlighted the superior performance of thinner layers with the Pt catalyst at an optimal operating temperature of 200 °C, albeit with incomplete recovery due to the strong binding energy of NO_2_ molecules. While higher temperatures facilitated full recovery, this came at the cost of reduced sensor responsiveness. Additionally, the study explored the significant impact of moisture in terms of enhancing the responsiveness of Pt-NiO and Au-NiO sensors. The higher sensor response in humid conditions compared to dry air is attributed to the displacement of pre-existing water on the surface by NO_2_ molecules. The findings underscore the potential of this simple fabrication approach for producing NiO nanowires, offering a promising and scalable alternative for applications in gas sensing and beyond. Nonetheless, over repeated cycles, the response of the NiO sensors toward NO_2_ decreases, which can be attributed to residual gas molecules that may remain on NiO’s active sites after successive measurements. This decline is also reflected in the increase in initial conductance in air by the 11th week compared to the first day, suggesting that NO_2_ molecules with high adsorption energy remain partially adsorbed on the surface, contributing to a persistent high surface conductivity. This effect highlights the critical trade-offs in long-term sensor reliability, where maintaining high response and recovery over extended use remains challenging for gas monitoring.

## Figures and Tables

**Table 1:** XRD parameters corresponding to the (200) crystallographic plane of NiO.

Parameter	Value
θ (degree)	43.38
$2\theta_{\mathrm{ICCD}}$ (degree)	43.29
β (degree)	0.373
D (nm)	229
δ $(10^{12}$ $\mathrm{line}/m^{2}$)	19
ε $(10^{-3}$)	1.5
d (Å)	2.083
$d_{\mathrm{ICDD}}$ (Å)	2.088
a (Å)	4.166
$a_{\mathrm{ICDD}}$ (Å)	4.176

**Table 2:** Summary of reported NO_2_ sensors based on metal oxides prepared by thermal oxidation.

Sensing Material	Morphology	Gas Concentration/Gas Response	Working Temperature
NiO (Present work)	NWs + NPs	5 ppm/G = 8.65	200 °C
WO_3_ [48]	Nanorods	10 ppm/R = 1.16	225 °C
CuO [49]	NWs	100 ppm/R* = 1.58	250 °C
CuO [50]	Thin film	5 ppm/R = 0.463	25 °C
ZnO [51]	Bunch of NWs	100 ppm/R = 6.22	200 °C
ZnO [51]	Nanorods	100 ppm/R = 1.01	200 °C
ZnSe/ZnO [52]	Mesoporous microstructure (nanoflakes+ crystals)	5 ppm/R** < 7	200 °C
TiO_2_ [53]	Thin film	5 ppm/R** = 0.31	25 °C

Notes: (R): resistive response, S = R_g_ − R_a_/R_a_. (R*): S = R_a_/R_g_. (R**): S = R_g_/R_a_. (G): conductometric response, S = G_a_ − G_g_/G_g_.

**Table 3:** Review of the performance of NiO sensors prepared via different methods.

Synthesis Method	Morphology	Gas Concentration/Gas Response	Working Temperature
Thermal oxidation (Present work)	NWs + NPs	NO_2_: 5 ppm/G = 8.65	200 °C
Thermal oxidation [54]	Granular film	H_2_S: 5 ppm/R′ = 2	RT
Hydrothermal [55]	Nanosheets	H_2_: 150 ppm/R = 1.91	250 °C
Hydrothermal [56]	Nano-petal film	H_2_S: 500 ppm/I = 4	300 °C
Hydrothermal [57]	hexagonal nanosheets	NO_2_: 15 ppm/G = 0.39	RT
Co-precipitation [58]	NPs	NO_2_: 5 ppm/1.75 < R″ < 2.25	180 °C
Sputtering [59]	Granular film	H_2_: 50 ppm/R = 0.23	250 °C
Sputtering [60]	Thin film	NH_3_: 1000 ppm/R = 2.89	250 °C
Sputtering [61]	Thin film	HCHO: 0.5 ppm/R = 0.168	200 °C

Notes: R′ = R_a_ − R_g_/R_a_. R″ = R_a_ − R_g_/R_g_. I = I_g_ − I_a_/I_a_.

## Data Availability

The original contributions presented in this study are included in the article. Further inquiries can be directed to the corresponding author.
